# Chromatin Network Analyses: Towards Structure-Function Relationships in Epigenomics

**DOI:** 10.3389/fbinf.2021.742216

**Published:** 2021-10-27

**Authors:** Vera Pancaldi

**Affiliations:** ^1^ Centre de Recherches en Cancérologie de Toulouse (CRCT), Institut National de la Santé et de la Recherche Médicale (Inserm) U1037, Centre National de la Recherche Scientifique (CNRS) U5071, Université Paul Sabatier, Toulouse, France; ^2^ Barcelona Supercomputing Center, Barcelona, Spain

**Keywords:** chromatin networks, nucleome, epigenomics, variability, structure-function, complex networks, Hi-C

## Abstract

Recent technological advances have allowed us to map chromatin conformation and uncover the genome’s spatial organization of the genome inside the nucleus. These experiments have revealed the complexities of genome folding, characterized by the presence of loops and domains at different scales, which can change across development and in different cell types. There is strong evidence for a relationship between the topological properties of chromatin contacts and cellular phenotype. Chromatin can be represented as a network, in which genomic fragments are the nodes and connections represent experimentally observed spatial proximity of two genomically distant regions in a specific cell type or biological condition. With this approach we can consider a variety of chromatin features in association with the 3D structure, investigating how nuclear chromatin organization can be related to gene regulation, replication, malignancy, phenotypic variability and plasticity. We briefly review the results obtained on genome architecture through network theoretic approaches. As previously observed in protein-protein interaction networks and many types of non-biological networks, external conditions could shape network topology through a yet unidentified structure-function relationship. Similar to scientists studying the brain, we are confronted with a duality between a spatially embedded network of physical contacts, a related network of correlation in the dynamics of network nodes and, finally, an abstract definition of function of this network, related to phenotype. We summarise major developments in the study of networks in other fields, which we think can suggest a path towards better understanding how 3D genome configuration can impact biological function and adaptation to the environment.

## Introduction

### The 4D Nucleome: Features and Dynamics of Chromatin Contacts

For just over 10 years we have had the tools to explore chromosome conformation inside the nucleus with Hi‐C (Lieberman-Aiden et al., 2009) and related techniques. Understanding the organization of the overall dynamic 3D structure of DNA in the nucleus with associated proteins and nucleic acids [the nucleome (Dekker et al., 2017)] remains an important step towards better understanding the connection between genome and phenotype. Beside the variations of Hi‐C [ChIA-PET (Fullwood et al., 2009), HiChIP (Mumbach et al., 2016), micro-C (Hsieh et al., 2015)], alternative approaches provide an independent picture of nuclear organization. For example, microscopy based techniques allow us to visualize previously tagged chromatin regions for inference of 3D interactions [DNA FISH Oligopaints (Beliveau et al., 2014), Hi-M (Gizzi et al., 2019)]. Moreover, methods that infer 3D proximity by segregating genomic fragments in different locations in the nucleus permit the inference of 3D contact maps independent of proximity ligation [GAM (Beagrie et al., 2017), SPRITE (Quinodoz et al., 2018; Quinodoz et al., 2020)], which is the traditional process to identify chromatin contacts by most Hi‐C and derived methods. Finally, imaging integrated approaches including RNA and chromatin mark quantification in single cells (Takei et al., 2021) are shedding an unprecedented light on single cell nucleome organization.

Given the large regions of the genome that do not contain genes, which are often the focus of our attention, variations of Hi‐C that allow us to study interactions involving a specific subset of chromosomal regions such as Capture Hi‐C have been particularly useful. In particular promoter capture Hi‐C (PCHi‐C) (Schoenfelder et al., 2015a; Mifsud et al., 2015) confirmed the existence and importance of long-range chromatin interactions and identified the role of important regulators of developmental genes, such as the Polycomb complex, in creating a highly interconnected core of genes in mouse embryonic stem cells (Schoenfelder et al., 2015b). Importantly, PCHi‐C allows us to study long-range interactions that can only be captured by Hi‐C using extremely deep sequencing.

The increased availability of chromatin conformation data has enabled detailed analysis of chromatin rearrangements during differentiation (Bonev et al., 2017), progression to malignancy (Malod-Dognin et al., 2020; Vilarrasa-Blasi et al., 2021), senescence (Chandra et al., 2015; Sati et al., 2020) and even during the cell cycle (Nagano et al., 2017; Zhang et al., 2019). Many results have been achieved with extensive use of mathematical models including polymer models, which assume DNA to be a polymer with specific properties that can be related to epigenomic modifications. As summarised in a recent review (Di Stefano et al., 2021), mainly two modelling approaches, a data-driven one and a bottom-up one starting from principles of polymer behaviour, have enabled exploration of dynamics at different scales. Single locus-scale dynamics was modelled in great detail, describing specific processes involved in gene activation and related chromatin changes. On the other hand, coarser models can describe large-scale chromatin rearrangements, compaction and decompaction events and movements to or from the nuclear lamina.

## Relating Chromatin Structure to Transcription and Replication

There is some evidence of a tight connection between chromatin contacts and transcription. More specifically, changes in the 3D looping structures of chromatin in neurons following their activation have been shown to affect gene expression on different time scales (Beagan et al., 2020). Also during reprogramming of mouse B cells into pluripotency, specific open chromatin 3D enhancer structures (network hubs) around active genes were shown to form in association to gene expression activation and disappear during gene silencing (Di Stefano et al., 2020). Interestingly, microscopy based techniques have also investigated chromatin dynamics and showed a caging effect that restricts chromatin motion in the proximity of a transcribed gene (Germier et al., 2017), suggesting that chromatin dynamics at various scales is closely associated with transcriptional regulation (Shaban et al., 2020). Whether structure drives the activation of transcription in gene assemblies (transcription factories) (Iborra et al., 1996), or mRNA production reshapes chromatin structure (Hilbert et al., 2021), it is clear that structure and transcription are tightly intertwined. Nevertheless a lack of a clear relation between transcription activation and 3D contacts, at least at single-cell level (Espinola et al., 2021; Ing-Simmons et al., 2021) leaves us with open questions.

On a larger scale, the role of typical structural features, such as Topologically Associating Domains (TADs), in gene expression remains hotly debated (Cavalheiro et al., 2021), with evidence that in some specific loci, perturbing TAD structure can lead to substantial changes in expression patterns, even related to pathology and morphological aberrations (Lupiáñez et al., 2015), while at the genome wide level, expression programs can be resilient to drastic chromosomal rearrangements (Ghavi-Helm et al., 2019).

At a whole nucleome scale, the formation of TAD cliques (highly interconnected groups of TADs) towards the nuclear lamina has been associated with differentiation (Paulsen et al., 2019) and large TAD-level rearrangements have been observed also during reprogramming towards pluripotency (Di Stefano et al., 2020).

Topological analysis of the chromatin network has also led to the identification of drastic rearrangements involved in the progression from healthy to malignant cells. Despite the added complications of considering genomic alterations that are characteristics of cancer cells, clear 3D rearrangements have been observed to accompany oncogenic transformation in B cells, beyond those encountered in normal B cell differentiation (Malod-Dognin et al., 2020; Vilarrasa-Blasi et al., 2021). Exploring nucleome dynamics in differentiation of T cells, it was proposed that some of these rearrangements can ensure the irreversibility and stability of the differentiation status (Hu et al., 2018). Recent results mapping chromatin architecture in neurons suggest the importance of modifications in the TAD structure in neuronal function. More specifically, specific TAD melting events involving particularly long genes important for neuronal functions are up-regulated by local chromatin decompaction (Winick-Ng et al., 2020).

Whereas the concept of a relationship between 3D genome structure and transcription has been controversial and has not been elucidated in the details, it has been known for decades that loops of chromatin are involved in replication and in the formation of replicons, held together by cohesin (Edenberg and Huberman 1975; Guillou et al., 2010). It has also been clear for years that TADs can be seen as units of replication timing (RT), and replication timing domains are clearly connected to phenotypic states and cell types (Ryba et al., 2011; Pope et al., 2014; Boulos et al., 2015), suggesting a strong connection between 3D structure and replication acting on larger scales than those of replicons. Recent work featuring the integration of replication datasets with chromatin contact maps reinforces these findings (Jodkowska et al., 2019; Marchal et al., 2019) and suggests that chromatin might be predominantly organised in structures that ensure specific replication programs, which might become the substrate of transcriptional organization. More specifically, we have found that regions with similar replication timing are close to each other linearly but also in 3D through long range (tens of megabase) interactions (Madrid-Mencía et al., 2020) and we have also observed clustering or chromatin regions containing origins with the same efficiency of activation (effectively the number of cells that use specific origins at any division) across large distances (Jodkowska et al., 2019). Recent reports of the effect of knocking out a main replication regulator, Rif1, have highlighted its possible impact in shaping RT in the entire genome and even an effect on epigenetic marks and organization (Klein et al., 2021). These results have started shedding light on the mechanistic connection between replication, 3D structure and the epigenomic state of the cell.

## Variability, Cellular Environment and Epigenomic Rearrangements

Genes that are variable (across single cells) are also more strongly regulated (along time-courses) and more evolvable (across species) (Ciliberti et al., 2007; Lehner 2008; Tirosh et al., 2009; Sigalova et al., 2020). Inter-individual non-genetic variability is of fundamental relevance in medicine and recent studies have highlighted its importance also in healthy individuals (Chen et al., 2016; Ecker et al., 2017). Chromatin is the common substrate that unifies these three different biological processes and we have proposed that the chromatin context, including local and global conformation, could be considered as one of the important factors affecting variability in methylation, gene expression and ultimately phenotype (Ecker et al., 2018). At a local level, it has been shown that the chromatin state can impact gene expression variability. For example, genes repressed by binding of the Polycomb complex, were shown to have more variable expression levels across single cells (Kar et al., 2017). Moreover, promoter features defining specific genomic characteristics of transcriptional start sites (promoter architecture) were identified to be the main determinants of expression variability across single drosophila embryos and across individuals in specific human tissues (Sigalova et al., 2020). Indeed expression variability across individuals was also predictive of differential expression after genetic perturbations or changes in environmental conditions. Promoter features that were found to be important in determining variability include the narrow or broad transcription initiation region, Transcription Factor (TF) occupancy or increased regulatory complexity. Since chromatin properties and TF binding patterns are highly correlated with 3D chromatin interactions, it can be speculated that groups of genes that have similar values for these features would be forming preferential contacts in 3D.

**FIGURE 1:**
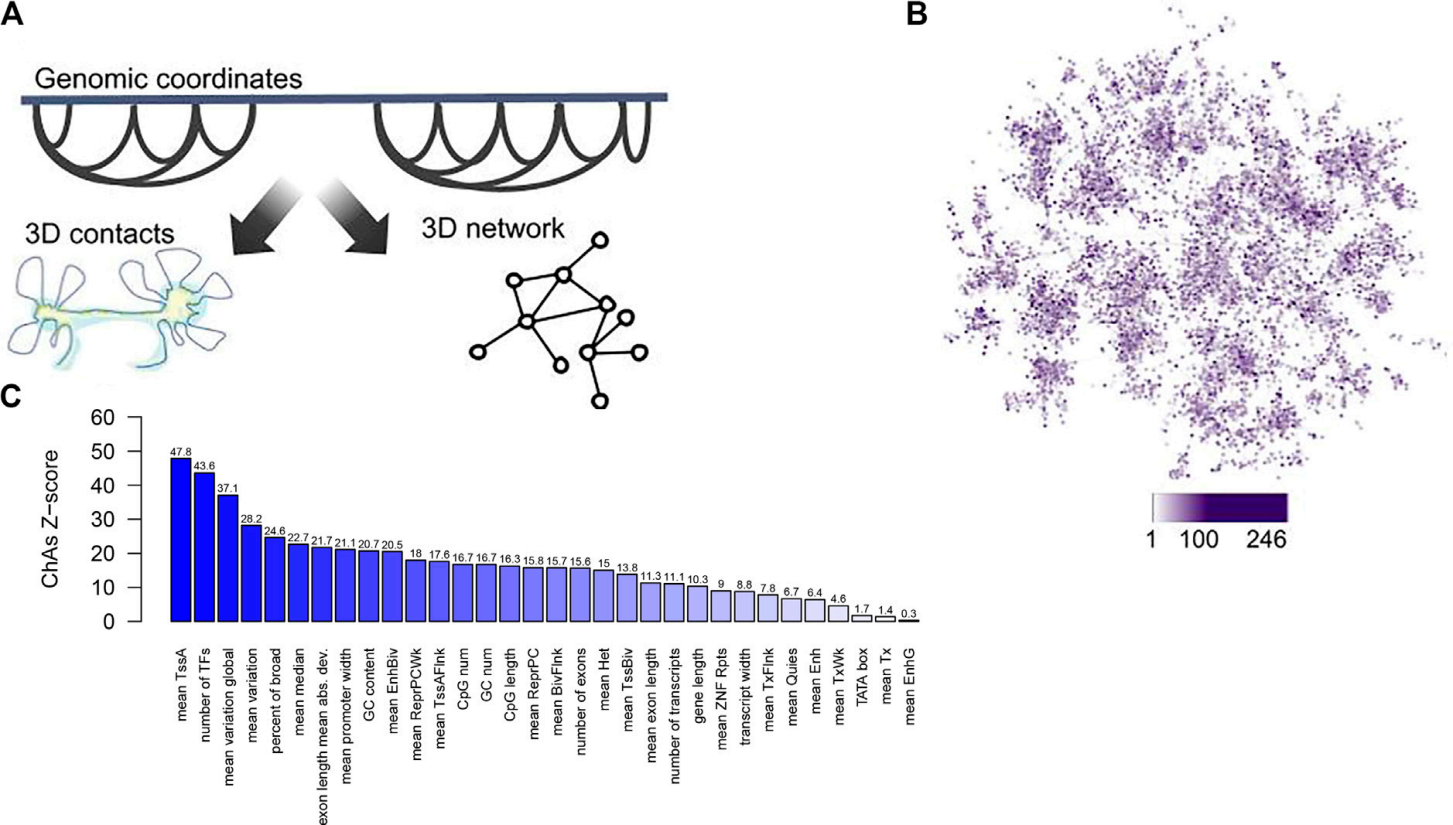
A chromatin assortativity analysis performed on the chromatin network shows preferential interactions of genes that have similar promoter characteristics. **(A)** Schematic describing the chromatin network approach in which nodes are chromatin fragments and edges describe experimentally identified 3D contacts between chromatin fragments, **(B)** Projection of promoter width from Sigalova et al. (2020) averaged across many tissues onto the combined Promoter Capture Hi‐C network for haematopoietic cells (Javierre et al., 2016). In this network nodes are gene promoters and node colour represents values of “promoter width” with increasing width shown as darker purple. **(C)** Chromatin assortativity analysis here measures whether promoters with similar values of the promoter features detailed in Sigalova et al. interact preferentially in the network from **(A)**. For each feature a ChAs value is calculated and the values obtained in 100 randomized networks is used to estimate a Z-score, shown on the barplot for each feature. The ChAs calculation is performed using distance preserving randomizations with the ChAseR package (Madrid-Mencía et al., 2020). High values of the Z-score denote preferential 3D distal interactions of promoters with similar values for these features.

Regions of the genome bound by Polycomb were found to cluster in 3D (Schoenfelder et al., 2015b; Pancaldi et al., 2016), as well as super-enhancer regions (data not shown), suggesting that the folding of chromatin in the nucleus might generate “areas” of higher or lower gene expression variability. This tendency for specific genomic regions to form clusters in 3D can be quantified using Chromatin Assortativity, which measures the significance of correlation between values of a feature on a chromatin region and other regions that interact with it (Pancaldi et al., 2016; Madrid-Mencía et al., 2020) against random expectation. Chromatin assortativity of the promoter features from Sigalova et al. (2020) projected onto a promoter interaction network for human cells derived from chromosome capture experiments indeed shows clustering of promoters of genes with similar sequence and transcriptional features in the 3D genome (Figure 1).

So far, we have described how specific regions of the genome can potentially be identified in 3D as having particular characteristic profiles of expression variability. Therefore we can wonder if changes in the external conditions of the cell might affect these nuclear organization patterns to promote adaptation. For example, strong evidence exists for an increase of overall expression and phenotypic variability when cells are grown under stressful environments (López-Maury et al., 2008) or in cancer (Brock et al., 2009) which involves strong regulation of response genes. Looking at a more global level, the mechanisms that control cellular variability in adaptation to external conditions could be reflected as rearrangements in chromatin organization. Indeed, the external environment impacts the organization of chromatin in the nucleus in space and its dynamics in time (the 4D nucleome). This is true across species and contexts, starting from changes of polycomb occupancy during heat-shock in drosophila (Li et al., 2015), to activation of transposable elements associated to chromatin decompaction in heat-stressed plants (Sun et al., 2020), to chromatin architecture rearrangements after osmotic stress in yeast (Amat et al., 2019) and during quiescence (Guidi et al., 2015).

Fibroblasts at late passages also showed telomeres shortening and shifting towards the nuclear center (Bridger et al., 2000), while differentiation, pluripotency and senescence also lead to widespread chromosomal rearrangements and drastic epigenomic changes (Bonev et al., 2017; Sati et al., 2020; Chovanec et al., 2021). These findings reinforce the concept that chromosomal organization is tightly related to cellular phenotype and function, which are strongly connected to the cell’s environment, opening the interesting question of which one between chromatin structure and phenotype is the cause or consequence.

We can therefore hypothesize the existence of a relationship between the global epigenomic characteristics of a cell population, especially 3D chromatin structure, and its plasticity in responding to changing environments or stimulation. To reveal this connection we are in need of a unified framework to represent the epigenome in an integrated fashion and with multiscale resolution.

One of the main challenges in modelling the epigenome is the lack of a complete understanding of the physics of chromatin (intended as DNA and all associated proteins, RNAs and modifications) and the reconciliation of different techniques to generate experimental data which are all affected by specific biases (for example, sequencing biases for Hi‐C related techniques, proximity/contact detection in microscopy based techniques) (Fiorillo et al., 2021). Moreover, we lack tools proposing integrated models covering different time and space scales. Network approaches to chromatin structure can provide a unified multi-scale picture of genome organization, while offering metrics for the overall characteristics of the 3D epigenome, to potentially relate them to phenotypes.

## Chromatin Network Approaches

The concept of using network theory to interpret chromatin structure datasets has been around for a decade. As soon as Hi-C contact maps were generated, they were interpreted as distance matrices and easily transformed into adjacency matrices of chromatin networks (Botta et al., 2010; Boulos et al., 2013; Boulos, 2014; Babaei et al., 2015; Morlot et al., 2016; Boulos et al., 2017). With increasing resolution and new chromosome capture techniques more chromatin network approaches were proposed (Pancaldi et al., 2016; Thibodeau et al., 2017; Norton et al., 2018; Huang et al., 2019; Malod-Dognin et al., 2020; Chovanec et al., 2021). The increasing interest in this framework has also prompted the development of 3D genome network visualizers, which allow users to explore the topology (contact patterns) of genomes in a non-linear fashion (Thibodeau et al., 2016; Madrid-Mencía et al., 2020; Ramirez et al., 2020; Chovanec et al., 2021) as well as to visualize other chromatin datasets in a 3D context. This approach can be considered complementary to 3D visualization of constraint based and polymer models of chromatin (Dekker et al., 2013; Lesne et al., 2014; Paulsen et al., 2017; Fiorillo et al., 2021).

In 2012 a pioneering paper by Sandhu et al. (2012) proposed an interesting analysis of the chromatin networks evinced by RNA Polymerase 2 ChIA-PET, a technique which provides 3D contacts between chromatin fragments mediated by polymerase. Despite the incompleteness of the obtained network, they merged different chromosome fragments within specific genomic regions to recover a connected network of promoters from the data set. Detailed analysis of this network’s topology highlighted communities related to specific biological functions and the presence of rich club nodes (highly connected nodes with other high degree nodes), representing important cellular processes, and less connected nodes (spokes), with specific developmental functions and which are enriched for genomic mutations and genetic polymorphisms. These results suggested that evolution has shaped the 3D genome structure.

More recently, similar network topology analyses applied to Hi‐C data targeted at investigating smaller regions within the genome (meso-scale) have identified the presence of core-periphery structures in certain TADs and suggest that the location of single nucleotide variants within these two regions of the TAD can determine the impact the variants have on diseases (Huang et al., 2019).

Network representations are particularly useful when using PCHi‐C datasets. As we have previously shown, promoter capture Hi‐C datasets in mESCs can be easily represented as networks involving promoters and other genomic regions (Other ends or Promoter Interacting Regions) generating networks which feature a main large connected component and various other smaller ones (Pancaldi et al., 2016). Despite not having time-resolved or single-cell chromatin contact networks, we used two statistical network properties (betweenness centrality and bridgeness) to estimate whether nodes with different chromatin marks were highly connected simultaneously with all their partners or with one at a time, akin to the definition of date and party hubs in PPIs (Han et al., 2004). Using these and other network statistics, we established that Polycomb mediated interactions are to some extent more stable and fixed, across cells or time (party hub). On the contrary, RNAPII was predicted to have more variable interactions, changing its partners across cells or time (date hub). The tools that we have produced (the ChAseR package and GARDEN-NET website) allow seamless integration and analysis of any property defined along the genome (gene properties such as transcriptional levels or variability, functional categories or evolutionary characteristics, chromatin features, or any user-defined data) onto the chromatin structures defined by any experimental chromatin contact datasets (Madrid-Mencía et al., 2020).

Importantly, networks allow us to visualize chromatin at different scales. Since TADs are defined similarly to network modules, namely as domains that have more interactions within them than with other domains, we can imagine grouping chromatin fragments within a TAD into a single node and investigating a network of TADs, leading to a hierarchical representation of the genome (Jodkowska et al., 2019). At the same time we can combine different datasets (for example Hi‐C and PCHi‐C) in a single network, to preserve a high number of long-range contacts while also considering the linear proximity of regions along the genome (Malod-Dognin et al., 2020).

Chovanec et al. (2021) presented a network approach to visualize and study the difference in 3D chromatin conformation between naive and primed human pluripotent stem cells (PSCs) using Canvas, a tool to represent chromatin fragment interaction networks in a multi-scale framework. They identified cell type specific clusters displaying coordinated gene expression and corresponding in some cases to functional units. Interestingly, they showed that TAD border insulation (separation between TADs) is stronger in primed compared to naive PSC, suggesting a coupling between phenotype and structure that would see naive cells with a “weaker” TAD structure. They suggested that smaller communities would come together into larger domains during the priming process, which is compatible with the formation of more functionally constrained units relying on long-range (>1 MB) polycomb enriched interactions between developmentally related genes.

Interestingly, the application of network theory analyses to chromatin allows us to peer into the organizing principles that underlie the nucleome in search for a structure-function relationship in chromatin networks.

## Chromatin Structure-Function Relationships: Multi-Disciplinary Concepts Towards an Epigenome-Phenotype Connection

Applying network theory approaches to biological networks has provided insight on different aspects of biological processes, from evolution and the structure of protein-protein interaction networks, all the way to mechanistic hypotheses on mode of action of drugs from looking at drug-disease-symptom interaction networks (Sonawane et al., 2019). The common theme through these approaches is the realization that a structure-function relationship exists between the topology and structural characteristics of a given network and the behaviour of the entity represented by that network.

To better examine the concept of structure-function relationships, we can turn to another biological entity that rivals the genome in complexity and mystery: the brain. Two separate approaches can be used to describe and study brain networks (Lynn and Bassett 2019). First, through imaging and tracing of water molecules along white matter, we can define a spatially embedded structural network connecting different regions of the brain. This approach is so far limited to a meso-scale level, involving the grouping of different neurons, but the latest technology can currently capture the dynamics of these meso-scale contacts in humans and primates. Second, we can take into account the functional relationship between different neurons, according to the principle that neural connections are strengthened when the two connected neurons fire together and that the emergence of collective firing behaviour of multiple neurons can give rise to cognitive functions. This approach, which relies on functional Magnetic Resonance Imaging (fMRI) allows us to identify correlation of activities between different nodes of the structural network (normally only possible at the brain region level including tens of thousands of neurons). A certain correlation between structural brain networks and functional brain networks is expected and identified, but the relation is certainly not trivial and the dynamics of the functional correlation might have to be considered (Suárez et al., 2020). These two networks and their relationship are specific to particular developmental stages and altered in mental disease.

**FIGURE 2:**
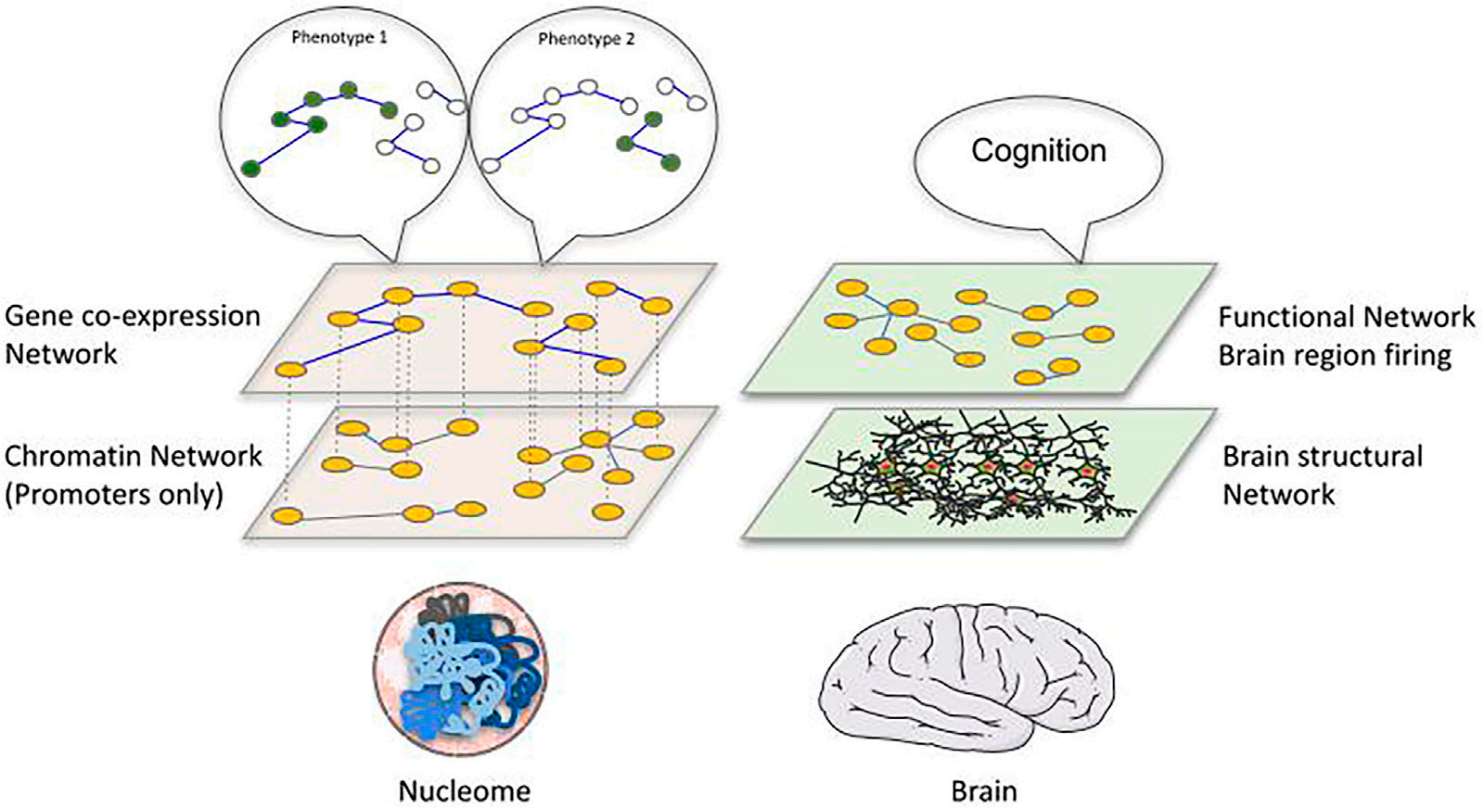
Proposing a structure-function relationship linking the epigenome to the phenotype. We propose an analogy between the brain and chromatin represented as multilayer networks. In the brain, a structural network of physical connections between neurons and brain regions can be identified and related to a functional network describing correlation in firing of distant brain regions, which collectively give rise to cognitive functions. Similarly, the physical interactions of genomic regions inside the nucleus **(left bottom layer:** chromatin network, nodes are gene promoters with contacts retrieved by promoter-capture Hi‐C) can be related to gene-coexpression networks **(left top layer:** nodes are genes and edges represent correlation of expression between them across different conditions) that ultimately determine cellular phenotypes, which can be interpreted as specific gene expression patterns associated to distinct phenotypes.

A correspondence between genome and brain networks becomes evident, with a parallelism between chromatin and brain structural networks versus co-expression and functional brain networks (Figure 2). Whereas in brain networks the clear connection between the two networks is represented by the signals exchanged between neurons, we are still discovering how the numerous molecular processes taking place inside a cell could provide a connection between 3D genome and phenotype. We could imagine the chromatin structure network and the gene/protein regulatory interaction or co-expression network as two components of a multilayer network (Kivelä et al., 2014), with phenotypes defined as an emergent collective behaviour on the gene co-expression network. A multilayer network has been successfully applied to the *C*. *elegans* connectome, showing the potential of such an approach in epigenomics (Bentley et al., 2016). A further important point of convergence between these two network types is their spatial character. More and more we are able to study chromatin structure through microscopy in single cells, providing us with spatial coordinates of genomic fragments. These new approaches allow us to go beyond the topology of the structure and create real maps of the genome inside each nucleus. A spatial perspective on chromatin could suggest exploiting a range of techniques that have been applied to other kinds of spatial networks, which normally focus on spreading phenomena, including mobility, epidemiology and power-grid networks, among many others (Barthélemy 2011). Such an approach in epigenomics would open new perspectives while requiring thorough considerations of the physics of the nucleus. To what extent an equivalence between spatial distance in chromatin and geographical distance is appropriate, together with the importance of transport on the chromatin network [information flow (Juan et al., 2016) or physical flow of molecules (Cortini and Filion 2018)] are likely to become topics of interest. A related issue that could have interesting repercussions in the epigenomics of diseases is the study of failures in these spatial networks and how resilient these networks are to attack (Schneider et al., 2011).

To further study this structure-function relationship and relate the cellular network to its environment, we can rely on results connecting network properties to their environment and behaviour. Multiple network metrics and topological features have been employed to describe networks’ response to specific conditions or perturbations, their resilience or plasticity (robustness or sensitivity) (Bar-Yam and Epstein 2004; Schneider et al., 2011; Csermely et al., 2015). Modularity in protein interaction networks has been attributed to the need for optimizing multiple tasks at once (Kashtan and Alon 2005) and was also observed in metabolic networks (Ravasz et al., 2002), again suggesting that structure in biological networks reflects environmental pressure. Particularly when looking at the topology of gene regulatory networks, changes in their architecture can be related to the cell’s environment (Luscombe et al., 2004).

In yeast, stress is associated with a differentiation of the expression programme, which can be reflected into a global rearrangement of the transcriptome associated with a drastic change of the protein interaction network. More specifically, heat stress produces a disaggregation in the interaction network of budding yeast (Mihalik and Csermely 2011) and we were ourselves able to show a similar effect in fission yeast (Lehtinen et al., 2013). We observed a decrease in module interconnectedness after stress treatment and measured a decrease in the overlap between network modules, representing a clear systemic disaggregation of the network topology. Interestingly, other approaches focussed on changes in metabolism before and after stress treatment arrived at similar conclusions using metabolic networks (Gopalacharyulu et al., 2009).

Some of these effects are reminiscent of phenomena that have been observed in social and ecological networks. In complex systems like social networks of traders in the stock market or schools of fish in the ocean, specific dynamics of synchronization have been related to success [prey evasion for fish or large profits for traders (Saavedra et al., 2011)]. Moreover, the structures of messaging networks (in which nodes are traders, edges represent instant messaging interactions across the day) were shown to be drastically affected by the situation of the stock market (Romero et al., 2016). In days of relative calm and predictable stock market fluctuations, traders exchange messages with all other traders, whereas on the days of strongly unpredictable price shocks the trader’s networks display a “turtled up” structure, with predominant connections to the network core. These are clear examples of relationships between network structure and its function in response to the environment.

In line with this view, it has been proposed that specific components in a biological network can have different functions, namely the core would serve to ensure a prompt response to known stimuli, while the periphery would contain elements needed to respond to unexpected situations (Csermely 2018). This interesting perspective implies that, on evolutionary timescales, repeated stimuli would lead to a change in the structure of the network corresponding to learning of the new conditions. Moreover, depending on the level of predictability of external conditions, the network might need to adapt its structure according to external conditions. These principles could govern the chromatin rearrangements that are observed in cells in response to changes in their external environment.

## Discussion

In summary, a large body of literature exists regarding system level analyses on protein-protein or gene-coexpression networks as well as networks from other disciplines. The notion that a cell’s phenotype is somehow encoded in its genome suggests that looking at the epigenomic networks under this light could also be relevant. When considering the 3D genome, Csermely’s hypothesis (Csermely 2018) might suggest that, through evolution, a core 3D network of highly conserved genes, involved in basic cellular functions, has been expanded by addition of new genes involved in new functions, specific to cell types and tissues. Importantly, changes in chromatin organization can thus encode changes in the cellular phenotype in response to varying needs of the cell to adapt to its external context. We anticipate that a network science perspective on (epi)genomes will continue to provide insights and new understanding for exploiting the rich datasets that will be produced across cell types, with potential applications in cancer and disease in general.

## Data Availability

Publicly available datasets were analyzed in this study. This data can be found here: https://www.embopress.org/action/downloadSupplement?doi=10.15252%2Fmsb.20209539&file=msb209539-sup-0002-Datasets.zip.
